# Impact of statin treatment on cardiovascular events in patients with retinal vein occlusion: a nested case-control study in Korea

**DOI:** 10.4178/epih.e2023035

**Published:** 2023-03-15

**Authors:** Joonsang Yoo, Joo Youn Shin, Jimin Jeon, Jinkwon Kim

**Affiliations:** 1 Department of Neurology, Yongin Severance Hospital, Yonsei University College of Medicine, Yongin, Korea; 2 Department of Ophthalmology, Yongin Severance Hospital, Yonsei University College of Medicine, Yongin, Korea; 3 Instutute for Innovation in Digital Healthcare, Yonsei University, Seoul, Korea

**Keywords:** Retinal vein occlusion, Myocardial infarction, Stroke, Cardiovascular diseases, Cohort studies

## Abstract

**OBJECTIVES:**

Retinal vein occlusion (RVO) is associated with an increased risk of future cardiovascular events. Statin therapy is a key cornerstone in prevention for patients at high cardiovascular risk. However, little is known about the role of statin therapy for patients with RVO. This study evaluated whether statin treatment in patients with RVO was associated with a lower risk of cardiovascular events.

**METHODS:**

A population-based, nested case-control study was conducted with a cohort of newly diagnosed RVO patients without prior cardiovascular disease between 2008 and 2020 using a nationwide health claims database in Korea. From this cohort of RVO patients, we identified cases of cardiovascular events (stroke or myocardial infarction) after RVO and matched controls based on sex, age, insurance type, antiplatelet use, and underlying comorbidities using 1:2 incidence density sampling.

**RESULTS:**

Using a cohort of 142,759 patients with newly diagnosed RVO, we selected 6,810 cases and 13,620 matched controls. A significantly lower risk of cardiovascular events (adjusted odds ratio, 0.604; 95% confidence interval, 0.557 to 0.655) was observed in RVO patients with statin treatment than in those without statin treatment. Statin treatment was associated with a reduced risk for both stroke and myocardial infarction after RVO. Longer statin treatment after RVO was associated with a lower risk for cardiovascular events.

**CONCLUSIONS:**

Statin treatment was associated with a lower risk for future cardiovascular events in patients with newly diagnosed RVO. Further studies are warranted to clarify the potential cardiovascular preventive role of statins in patients with RVO.

## GRAPHICAL ABSTRACT

[Fig f4-epih-45-e2023035]

## INTRODUCTION

Retinal vein occlusion (RVO) is a common vascular disorder involving partial or complete obstruction of the retinal vein and is one of the leading causes of vision impairment worldwide [1-3]. As the population continues to age, the incidence and associated burden of RVO have increased rapidly [4,5]. Epidemiological evidence has established that patients with RVO are at increased risk for the development of myocardial infarction (MI) or stroke [6- 11]. Both RVO and cardiovascular disease share common vascular risk factors, such as old age, smoking, hypertension, diabetes mellitus, and increased blood viscosity [12,13]. To mitigate the health and economic burden of cardiovascular complications, optimal strategies must be established to reduce cardiovascular events in high-risk patients with RVO.

Statins are one of the most commonly prescribed first-line drugs for lipid-lowering treatment and are an essential component of cardiovascular prevention. Since overwhelming evidence supports that statins efficiently reduce cardiovascular events, statin therapy is strongly recommended for those with or at high future risk for cardiovascular disease [14-16]. Considering the underlying pathogenesis of thrombosis and frequent cardiovascular complications in RVO patients, statin treatment may effectively reduce the increased cardiovascular risk in such patients. Retinal artery occlusion, another type of acute retinal vascular occlusion that causes mono-ocular visual loss, is mechanically similar to an ischemic stroke, and statin treatment is generally recommended for patients with retinal artery occlusion to prevent future cardiovascular events [17]. However, the role of statin treatment in the control of cardiovascular risk after RVO remains unclear. This study evaluated whether statin treatment following RVO was associated with reduced risk for cardiovascular events based on the nationwide healthcare claims database in Korea.

## MATERIALS AND METHODS

### Data source

This study had a population-based nested case-control design and used the nationwide health claims database in Korea. Korea has a public and single-payer system covering the entire population (approximately 50 million people). The Health Insurance Review & Assessment Service (HIRA) is responsible for reviewing medical claims from healthcare providers and quality of care. HIRA collects health claims data from hospital visits, medical procedures, prescriptions, diagnoses, and demographic information, including the sex, age, insurance type (National Health Insurance and Medical Aid from the government), and death statistics of its participants [18,19]. At each hospital visit, diagnoses are recorded according to the 10th revision of the International Statistical Classification of Diseases (ICD-10) codes in the HIRA database. HIRA has constructed a healthcare big data system and has opened the database to public health researchers (http://opendata.hira.or.kr/). The HIRA dataset, which is restricted to academic or public research use, is fully anonymized and does not contain identifiable information.

### Study participants

From the HIRA database, we selected patients with a primary diagnosis of RVO (ICD-10 code: H34.8) between January 2007 and March 2020 [6,20]. The index date of this RVO cohort was defined as the date of the first diagnosis of RVO. To exclude chronic RVO patients diagnosed previously, those with a diagnosis of retinal vascular disease (H34) or exposure to anti-vascular endothelial growth factor during the washout period (2007) were excluded. We also excluded RVO patients aged < 20 years and those with prior cardiovascular disease (ischemic heart diseases: I20-I25; stroke: I60-I64, I69, carotid artery stent, carotid endarterectomy, coronary stent insertion, coronary artery bypass graft) before the RVO diagnosis. Patients followed up for < 1 month because of an early outcome or censoring were also excluded because assessing the treatment effect with statins is challenging in patients whose cardiovascular events developed concurrently with RVO or immediately after RVO.

### Primary outcome

Patients were followed from the index date of the RVO diagnosis until the development of the primary outcome, loss of participant eligibility, death, or June 30, 2021 (study end date), whichever occurred first in the HIRA database. The primary outcome was defined as a composite of stroke or MI after RVO. Stroke was defined as an admission to the hospital for at least 3 days with a primary diagnosis of I60-I63 and having undergone brain computed tomography or magnetic resonance imaging during the admission period in an effort to only include patients with acute stroke [21,22]. MI was defined as admission with a primary diagnosis of I21 [23]. The diagnostic accuracy for stroke and MI based on the health claims data in the HIRA database was reported to be sufficient (> 80%) in prior validation studies [24,25].

### Nested case-control design

To construct a nested case-control study, we defined cases as patients with a primary outcome event (stroke or MI) during the follow-up period after RVO. For each case, we sampled 2 controls from the cohort by incidence density sampling [26]. Based on the dynamic risk set at the time of case occurrence, controls were selected with replacement from all persons who were event-free and at risk at the time of case occurrence, excluding the case itself (Supplementary Material 1). The controls were matched by same sex, age (± 1 year) at the diagnosis of RVO, insurance type, follow-up time from the index date, presence of comorbidities (hypertension, diabetes mellitus, atrial fibrillation, renal disease, and malignancy), use of antiplatelets before RVO, and treatment with antiplatelets at the time of outcome occurrence of the case.

### Exposure to statins and antiplatelets

In Korea, statins are prescribed by physicians at hospitals. Therefore, the prescription data (drug name, duration) for statins (atorvastatin, fluvastatin, lovastatin, pitavastatin, pravastatin, rosuvastatin, and simvastatin) are available from the HIRA database. Based on the prescription data, the use of statins before RVO was determined by exposure to the medications within the past 7 days from the index date of RVO diagnosis. Treatment with medication during the follow-up period after RVO typically had time-varying characteristics [27]. In this nested case-control study, treatment with statins was determined according to whether the patients had exposure to statins within the past 7 days when the case developed the primary outcome and the matched time in controls. The cumulative exposure to statins after RVO was calculated as the sum of days covered by statins between the RVO diagnosis and time of the primary outcome in cases or the matched time in controls, subdivided into 4 categories: ≤ 90 days, 91 days to 1 year, 1-2 years, and > 2 years. Along with statins, we also evaluated the pre-RVO use of antiplatelets (aspirin, clopidogrel, ticlopidine, ticagrelor, prasugrel, triflusal, dipyridamole, and cilostazol) and treatment with antiplatelets after RVO.

### Covariates

We collected data on sex and age at the time of RVO diagnosis. The presence of comorbidities (hypertension, diabetes mellitus, atrial fibrillation, renal disease, and malignancy) was determined by the health claims data in the HIRA database [28]. Hypertension and diabetes mellitus were considered relevant if patients received anti-hypertensive agents (calcium-channel blockers, angiotensin-converting enzyme inhibitors, angiotensin-receptor blockers, diuretics, beta-blockers, alpha-blockers, and/or vasodilators) or anti-diabetic agents (sulfonylureas, biguanides, alpha-glucosidase inhibitors, thiazolidinediones, meglitinides, glucagon-like peptide-1 receptor agonists, dipeptidyl peptidase-4 inhibitors, and/or insulin) and had the corresponding diagnostic codes (hypertension: I10-I15, diabetes mellitus: E08-E11 or E13-E14). Atrial fibrillation was identified by the presence of the I48 code. Renal disease was determined by the presence of relevant diagnostic codes (N17-N19, E082, E102, E112, E132, or I12-I13) or claims for hemodialysis, peritoneal dialysis, and/or procedures or prescriptions related to renal disease [28]. Malignancy was determined as the presence of a diagnostic code of malignancy (C00-C97) and a cancer-specific insurance code (V027, V193, and V194), which is assigned to provide economic benefits to confirmed cancer patients [29]. In this nested case-control study, the presence or absence of comorbidities was investigated up to the time of occurrence of the primary outcome in cases or the matched time in controls.

### Statistical analysis

The baseline characteristics were summarized as the number of patients (%) for categorical variables and mean± standard deviation values for continuous variables. Using the matched case-control data, we performed conditional logistic regression to estimate the odds ratio (OR) and 95% confidence interval (CI) for the treatment effect of statins on the primary outcome. Multivariable conditional logistic regression included the year of RVO diagnosis, use of statins before RVO, and treatment with statins after RVO. We also investigated the risk for the primary outcome according to the cumulative exposure to statins.

Individual regression models for each outcome component (ischemic stroke: I63, hemorrhagic stroke: I60-I62, and MI: I21) were created for secondary outcome analyses. We performed subgroup analyses according to sex, age, the presence of hypertension, and diabetes mellitus. We also performed a subpopulation analysis according to the year of RVO diagnosis. E-value analysis was conducted as a sensitivity analysis to assess the robustness of the association between statins and primary outcome. The E-value is an estimate of the minimum strength of unmeasured confounding needed to fully explain away the observed association between the exposure and the outcome [30]. Smaller E-values suggest that little unmeasured confounding could generate the effect estimate [31]. To better understand the association between statin treatment after RVO and the risk of the primary outcome, an event-free survival plot in the RVO cohort was illustrated using the Simon & Makuch [32] method, which is an expansion of the Kaplan-Meier plot with respect to a time-dependent variable. All statistical analyses were performed using SAS version 9.4.2 (SAS Institute Inc., Cary, NC, USA), and R version 3.5.1 (R Foundation for Statistical Computing, Vienna, Austria). A p-value <0.05 was considered statistically significant.

### Ethics statement

This study was approved by the Institutional Review Board of Yongin Severance Hospital, Yonsei University College of Medicine (9-2020-0116), and the requirement for informed consent was waived because this study conducted a retrospective analysis using fully anonymized data. The study was performed in accordance with approved guidelines and regulations for medical research expressed in the Declaration of Helsinki.

## RESULTS

### Cohort of retinal vein occlusion

**Figure 1.**
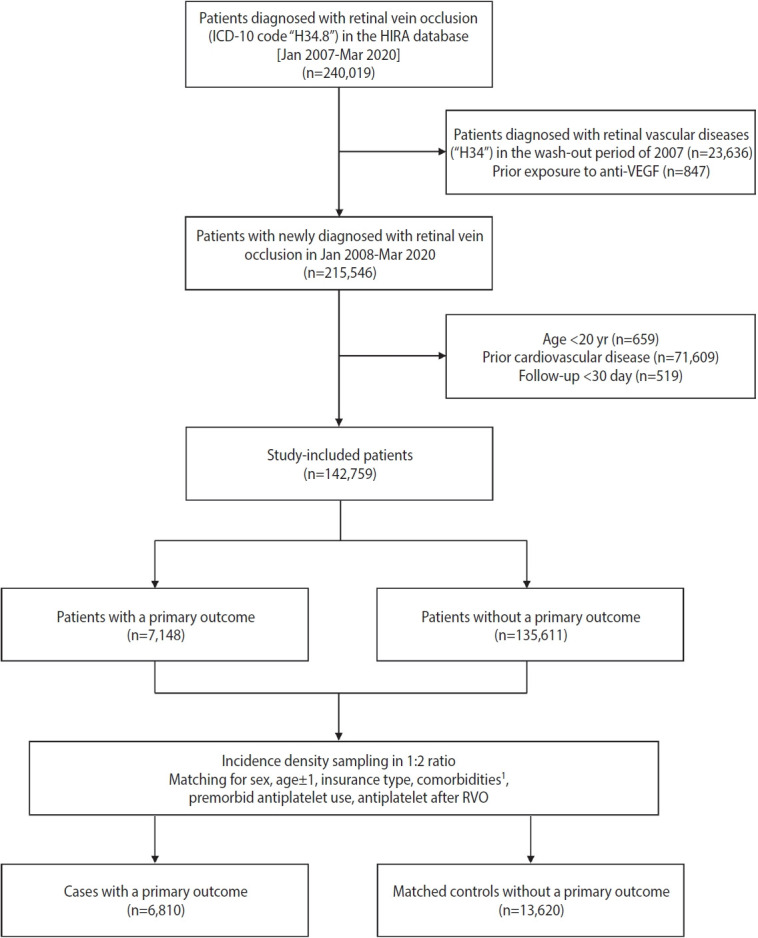
Flowchart of the selection of study participants according to inclusion and exclusion criteria. HIRA, Health Insurance & Review Assessment; ICD-10, International Classification of Diseases, 10th revision; RVO, retinal vein occlusion; VEGF, vascular endothelial growth factor. ^1^Comorbidities: hypertension, diabetes mellitus, atrial fibrillation, malignancy, and renal disease.

During the study period, 240,019 patients were diagnosed with RVO. According to the inclusion and exclusion criteria, a cohort of 142,759 patients with newly diagnosed RVO and no prior cardiovascular disease was identified (Figure 1). The mean age at RVO diagnosis was 60.24± 12.64 years, and 44.95% of patients were male (Supplementary Material 2). Of the 142,759 RVO patients, 48,348 (33.87%) were diagnosed between 2008 and 2011, 44,339 (31.06%) were diagnosed between 2012 and 2015, and 50,072 (35.07%) were diagnosed between 2016 and 2020. Among the RVO patients, the proportion of patients with premorbid use of statins was 14.24%. We also assessed the proportions of patients who received statins throughout the follow-up period after the index date of RVO (Supplementary Material 3). Immediately after the index date, an abrupt increase was found in patients who received statin treatment. Throughout the long-term follow-up period, the proportion of patients who received statins gradually increased.

### Cardiovascular outcomes by statin treatment after retinal vein occlusion

**Figure 2.**
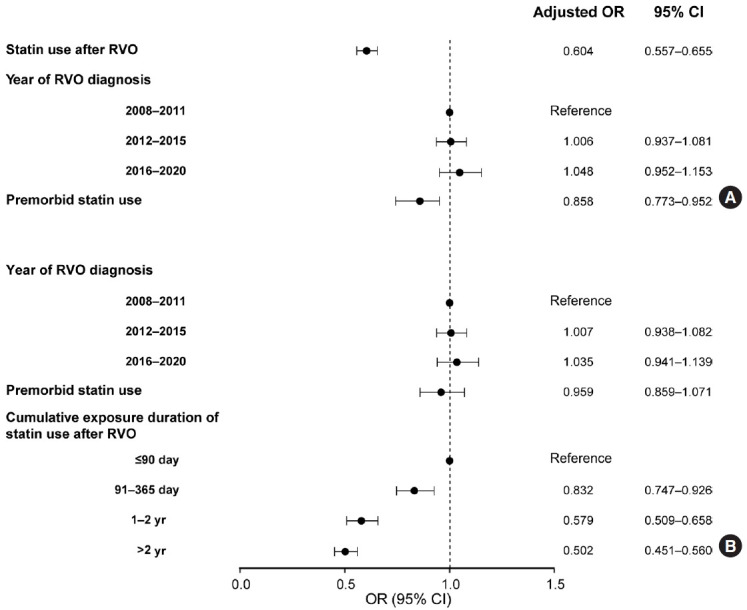
Effects of statins on the risk for the primary outcome after RVO. The data were obtained from multivariable conditional logistic regression analysis with the case-control dataset matched for sex, age, insurance type, hypertension, diabetes mellitus, atrial fibrillation, renal disease, malignancy, use of antiplatelets before RVO, and treatment with antiplatelets after RVO. Adjustments were made for the variables listed in the figure. (A) Statin treatment after RVO. (B) Cumulative duration of statin exposure after RVO. RVO, retinal vein occlusion; OR, odds ratio; CI, confidence interval.

During the mean follow-up period of 6.69± 3.60 years after the diagnosis of RVO, 7,148 patients (5.01%) had a primary outcome. In the nested case-control design, 6,810 cases (95.27% of the patients with a primary outcome) were matched to 13,620 controls without a primary outcome using 1:2 incidence density sampling. Among them, the mean time from the RVO diagnosis to the primary outcome was 4.50± 3.15 years. The control group received statin treatment more frequently after RVO than the case group (29.82% in the control group and 20.94% in the case group; p< 0.001) (Table 1). In multivariable conditional logistic regression, statin treatment after RVO was significantly associated with a lower risk for the primary outcome (adjusted OR, 0.604; 95% CI, 0.557 to 0.655) (Figure 2A). The E-value for the point estimate was 2.70 and for the upper CI limit was 2.42. The premorbid use of statin was also related to lower cardiovascular risk (adjusted OR, 0.858; 95% CI, 0.773 to 0.952). Regarding the relationship with cumulative exposure to statins after RVO, cardiovascular events occurred less as the duration of statin exposure increased. Compared to cumulative statin exposure of ≤ 90 days, the adjusted OR 91-365 days was 0.832 (95% CI, 0.747 to 0.926), that for 1-2 years was 0.579 (95% CI, 0.509 to 0.658), and that for > 2 years was 0.502 (95% CI, 0.451 to 0.560) (Figure 2B). As shown in a cumulative incidence plot for the primary outcome in the RVO cohort of 142,759 patients, statin treatment was associated with a lower risk of the primary outcome after RVO (Supplementary Material 4).

### Secondary outcome analysis

Among 6,810 cases with the primary outcome, 5,519 patients had a stroke (ischemic stroke in 4,074 cases and hemorrhagic stroke in 1,445 cases) and 1,291 patients had an MI. In the secondary analyses for individual outcomes (Table 2), the risk reduction with statin treatment was significant for both stroke (adjusted OR, 0.563; 95% CI, 0.513 to 0.617) and MI (adjusted OR, 0.782; 95% CI, 0.657 to 0.932).

### Sensitivity analyses

**Figure 3.**
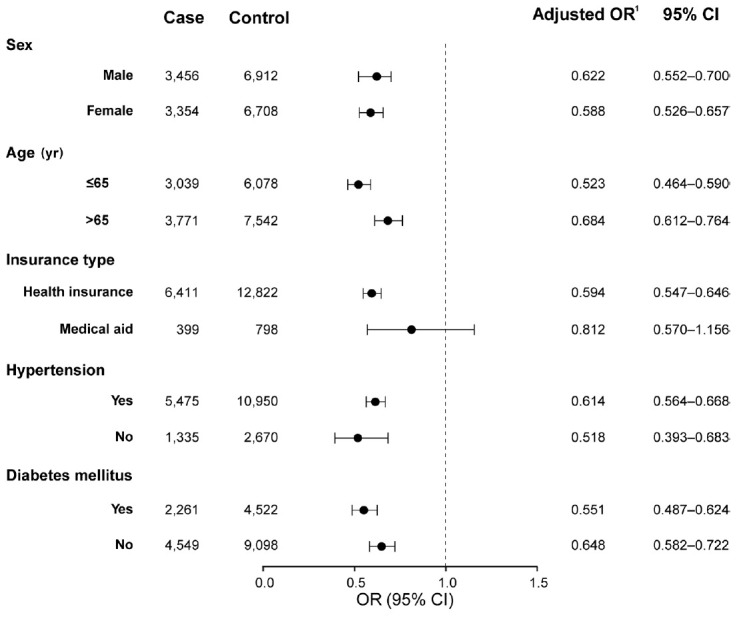
Results of subgroup analyses for the primary outcome according to statin treatment after retinal vein occlusion. Controls are matched with each case for sex, age, insurance type, hypertension, diabetes mellitus, atrial fibrillation, renal disease, malignancy, use of antiplatelets before retinal vein occlusion, and treatment with antiplatelets after retinal vein occlusion. OR, odds ratio; CI, confidence interval. ^1^Derived from multivariable conditional logistic regression adjusted for the year of retinal vein occlusion diagnosis, and premorbid use of statins.

As sensitivity analyses, we performed subgroup analyses according to sex, age, insurance type, and the presence of hypertension or diabetes mellitus and a subpopulation analysis according to the year of RVO diagnosis. In the subgroup analyses, the risk reduction with statin treatment was consistent regardless of sex, age, hypertension, or diabetes mellitus (Figure 3). Analyses of subpopulations according to the year of RVO diagnosis (2008-2011, 2012-2015, 2016-2020) revealed that the risk for the primary outcome was negatively associated with statin treatment and the duration of statin exposure after RVO across all study periods (Supplementary Material 5).

## DISCUSSION

Using a population-based cohort with RVO, we investigated whether treatment with statins following RVO was associated with a decreased risk of cardiovascular events. During the 6.69 years of mean follow-up, cardiovascular events occurred in 5.01% of the patients with RVO. About four-fifths of them developed a stroke, and the remaining one-fifth had an MI. In this nationwide, nested case-control study, RVO patients who received treatment with a statin had a lower incidence of subsequent cardiovascular events than those without statin treatment. The risk reduction was identified both for stroke and for MI. Additionally, a longer duration of statin exposure was correlated with a lower cardiovascular risk. The reduction of cardiovascular events with statin treatment was consistent regardless of the year of the RVO diagnosis.

RVO is a common ophthalmologic emergency leading to visual loss, and patients with RVO have an increased risk of a subsequent cardiovascular event such as stroke and MI [6-8,10,11]. Even with the well-established increased cardiovascular risk in RVO patients, no definitive guideline or recommendation exists regarding how to prevent secondary vascular events in RVO patients. Additionally, it remains unclear whether it is appropriate to use additional cardiovascular medications to prevent vascular events and, if so, which medications to use. Prior studies evaluating the effect of anti-thrombotic and fibrinolytic agents on patients with RVO demonstrated that medications improved visual acuity and prevented iris neovascularization [33,34]. However, long-term data on the efficacy of these medications as secondary vascular prophylaxis are lacking. A retrospective case-control study evaluated the effects of aspirin and statins on the visual outcomes in 42 high-risk patients with RVO [35]. Unfortunately, the study was underpowered and could neither show the benefits of aspirin or statins nor evaluate the long-term cardiovascular risk.

Although RVO is a disease of the retinal vein, the mechanism is hypothetically shared with arterial diseases such as stroke and MI. The exact mechanism of RVO development has not been yet fully elucidated, but likely involves a combination of 3 systemic changes known as Virchow’s triad: hemodynamic changes (venous stasis), degenerative changes in the vascular wall, and excessive blood coagulation [36-38]. Additionally, the retinal vein shares an adventitial sheath with the retinal artery, where arteriolosclerosis causes narrowing of thin-walled veins and stasis of venous return [36]. This mechanical compression and venous tortuosity can cause turbulent flow, endothelial cell stress, and thrombosis [3,37,39]. Systemic vascular comorbidities and a prothrombotic state can play a role in the development of RVO [3]. Prothrombotic conditions and traditional atherosclerotic risk factors are prevalent in patients with RVO [38,40]. The thrombus generated through this process blocks the retinal vein, resulting in RVO. In postmortem eyes with RVO, this thrombus was identified around the retinal vein [41].

In the present study, the use of statins was associated with a 40% reduced incidence of stroke or MI in patients with RVO. Statins are typically recommended for patients with established cardiovascular disease, as secondary prevention, and for those expected to have an increased risk of future cardiovascular disease, as primary prevention [42,43]. Lipid-lowering therapy with statins, supported by a broad evidence base, is the mainstay of cardiovascular risk reduction and prevention, as recommended by international guidelines [14,15]. It is also known that RVO risk and increased lipoprotein levels are related [44]. However, little is known about the preventive role of statins in RVO patients with a high risk of developing a stroke or MI [9,45]. Statins play a role in the stabilization and regression of atherosclerotic plaques by reducing the lipid content [46]. Beyond their lipid-lowering effect, statins have pleiotropic, cholesterol-independent effects associated with their anti-inflammatory, anti-thrombotic, blood viscosity-reducing, vasodilating, and endothelial protective properties [47]. They also have a vascular remodeling effect with immunomodulatory properties [48]. After pulmonary embolism, a type of venous thromboembolism, statin treatment reduced the risk for cardiovascular events and all-cause mortality, as well as recurrent pulmonary embolism [49]. Considering the pathophysiology of RVO and underlying risk factors, these beneficial effects of statins are expected to be sufficiently effective in preventing subsequent cardiovascular events in high-risk RVO patients.

Our data showed that the proportion of RVO patients taking statins increased throughout follow-up, likely caused by the increasing age of the patients and expansion of statin indications in recent years. However, regarding the beneficial effects of statins, the proportion of RVO patients taking statins seemed low, making it a potential treatment target for high-risk patients [43]. Although further research is needed, statin administration is recommended to start and continue for cardiovascular prevention in patients with RVO.

Since the study was conducted with a nationwide cohort, the study population with RVO was large, enabling long-term follow-up for the development of cardiovascular events in patients with RVO. However, this study had several limitations. Although we used a population-based cohort, it had a retrospective design; therefore, the residual confounding effect of uncollected variables is possible. Without an intervention, we could not draw a conclusion regarding the causality of the beneficial associations of statins in this observational study. Due to the lack of detailed clinical data in health claims, potentially important information such as smoking, physical activity, and severity of RVO could not be collected. Since the diagnosis of RVO is also based on diagnostic codes in health claims data, misclassification and validation are potential problems. Additionally, we could not analyze branch RVO and central RVO separately since the diagnostic codes were not differentiated. We also could not distinguish between ischemic and non-ischemic RVO. However, considering that a significant proportion of non-ischemic RVO cases are converted to ischemic RVO [1], the results of our study would be helpful in the overall management of patients with RVO. The development of cardiovascular events after RVO was identified based on the presence of an admission with the related diagnostic codes. Although prior validation studies in Korea have reported that the diagnosis of cardiovascular events according to these criteria was accurate, there may be a difference between the real occurrence of cardiovascular events and the risk determined based on health claims. A difference may also exist between the prescription history of a drug and patients’ actual drug-taking history. Furthermore, care should be taken in generalizing the results of this study to other countries because Korea is relatively ethnically homogeneous.

In conclusion, this population-based cohort study found that statin treatment was significantly associated with a lower risk of cardiovascular events in patients with newly diagnosed RVO. Further study is warranted to clarify the potential preventive role of statins in patients with RVO.

## DATA AVAILABILITY

The dataset used in this study is accessible from HIRA, but restrictions apply to the availability of these data, which were used under license for the current study, and so are not publicly available. The data are only available upon reasonable request of investigators for academic or political purposes and permission from the inquiry committee of research support in HIRA (https://opendata.hira.or.kr/or/orb/useGdInfo.do).

## Figures and Tables

**Figure f4-epih-45-e2023035:**
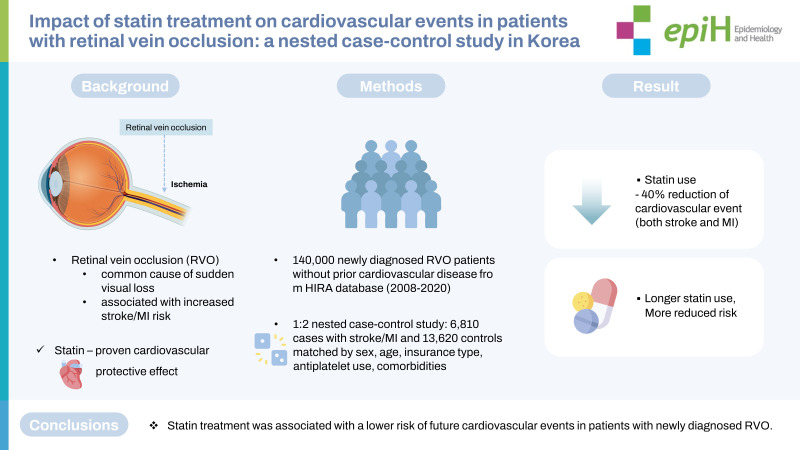


**Table 1.** Case and control patients with RVO

Variables		Case (n=6,810)	Control (n=13,620)	Crude OR (95% CI)^1^	p-value
Sex, male		3,456 (50.75)	6,912 (50.75)	Matched	
Age (yr)		65.69±11.27	65.66±11.23	Matched	
Year of RVO diagnosis					
	2008-2011	3,621 (53.17)	7,111 (52.21)	1.000 (reference)	
	2012-2015	2,118 (31.10)	4,326 (31.76)	0.954 (0.889, 1.023)	0.189
	2016-2020	1,071 (15.73)	2,183 (16.03)	0.951 (0.866, 1.045)	0.296
Insurance type					
	National Health Insurance	6,411 (94.14)	12,822 (94.14)	Matched	
	Medical Aid	399 (5.86)	798 (5.86)	Matched	
Comorbidity					
	Hypertension	5,475 (80.40)	10,950 (80.40)	Matched	
	Diabetes mellitus	2,261 (33.20)	4,522 (33.20)	Matched	
	Atrial fibrillation	439 (6.45)	878 (6.45)	Matched	
	Renal disease	972 (14.27)	1,944 (14.27)	Matched	
	Malignancy	458 (6.73)	916 (6.73)	Matched	
Premorbid use of medication					
	Statin	702 (10.31)	1,950 (14.32)	0.668 (0.607, 0.734)	<0.001
	Antiplatelet	900 (13.22)	1,800 (13.22)	Matched	
Time from RVO diagnosis to primary outcome (yr)		4.50±3.15	4.50±3.15	Matched	
Treatment after RVO					
	Statin	1,426 (20.94)	4,062 (29.82)	0.578 (0.536, 0.623)	<0.001
	Antiplatelet	1,417 (20.81)	2,834 (20.81)	Matched	
Cumulative statin exposure after RVO					
	≤90 day	4,867 (71.47)	8,525 (62.59)	1.000 (reference)	
	91-365 day	660 (9.69)	1,329 (9.76)	0.826 (0.745, 0.917)	<0.001
	1-2 yr	412 (6.05)	1,149 (8.44)	0.573 (0.507, 0.648)	<0.001
	>2 yr	871 (12.79)	2,617 (19.21)	0.494 (0.449, 0.544)	<0.001

Values are presented as number of patients (%) or mean±standard deviation. RVO, retinal vein occlusion; OR, odds ratio; CI, confidence interval.

1 Obtained from conditional logistic regression analysis.

**Table 2.** Risk for individual outcomes according to statin treatment after RVO

Outcome	Adjusted OR (95% CI)^1^			
	All stroke (n=5,519)	Ischemic stroke (n=4,074)	Hemorrhagic stroke (n=1,445)	Myocardial infarction (n=1,291)
Statin after RVO	0.563 (0.513, 0.617)	0.565 (0.509, 0.628)	0.555 (0.460, 0.671)	0.782 (0.657, 0.932)

The data are obtained from multivariable conditional logistic regression analyses using the case-control dataset matched for sex, age, insurance type, hypertension, diabetes mellitus, atrial fibrillation, renal disease, malignancy, premorbid use of antiplatelets before RVO, and treatment with antiplatelets. RVO, retinal vein occlusion; OR, odds ratio; CI, confidence interval.

1 Adjustments were made for the year of RVO diagnosis, and premorbid use of statins.

## SUPPLEMENTARY MATERIALS

Supplementary Material 1. Schematic design of 1:2 nested case-control design using incidental density sampling

Supplementary Material 2. Clinical characteristics of the study participants with retinal vein occlusion

Supplementary Material 3. Proportion of patients who had received statins after a diagnosis of retinal vein occlusion

Supplementary Material 4. Estimates of the cumulative incidence for primary outcome according to the use of statins after retinal vein occlusion

Supplementary Material 5. Association between statin treatment and cardiovascular risk in subpopulations by year of retinal vein occlusion diagnosis
