# Supplementary material for: Impact of statin treatment on cardiovascular events in patients with retinal vein occlusion: a nested case-control study in Korea
Source: Epidemiol Health. 2023 Mar 15;45:e2023035. doi: 10.4178/epih.e2023035 (PMC10396806; doi:10.4178/epih.e2023035)
Supplement: Supplementary Material 1. — Schematic design of 1:2 nested case-control design using incidental density sampling [file epih-45-e2023035-Supplementary-1.docx]

**SUPPLEMENTARY MATERIALS**

**Supplementary Material 1.** Schematic design of 1:2 nested case-control design using incidental density sampling

**
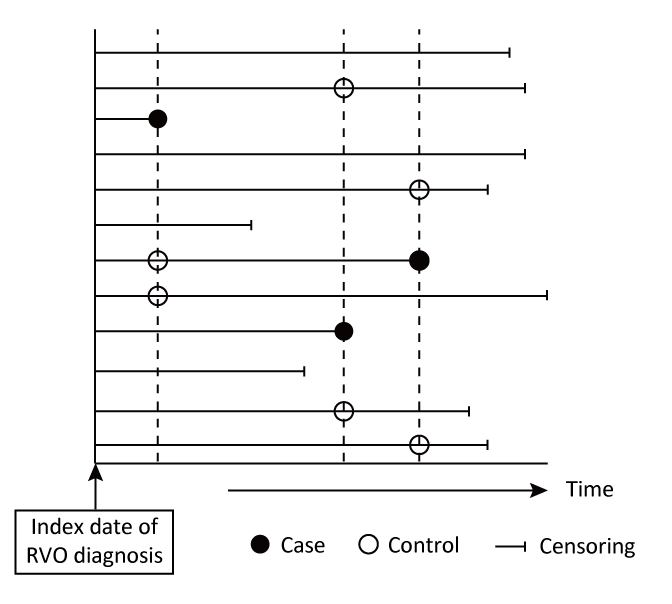
**

RVO, retinal vein occlusion.
