# Supplementary material for: Impact of statin treatment on cardiovascular events in patients with retinal vein occlusion: a nested case-control study in Korea
Source: Epidemiol Health. 2023 Mar 15;45:e2023035. doi: 10.4178/epih.e2023035 (PMC10396806; doi:10.4178/epih.e2023035)
Supplement: Supplementary Material 2. — Clinical characteristics of the study participants with retinal vein occlusion [file epih-45-e2023035-Supplementary-2.docx]

**Supplementary Material 2.** Clinical characteristics of the study participants with retinal vein occlusion

| Variable | Total (n=142,759) |
| --- | --- |
|  |  |
| Sex, male | 64,172 (44.95) |
| Age, years | 60.24 ± 12.64 |
| Insurance type |  |
| Health insurance | 135,698 (95.05) |
| Medical aid | 7,061 (4.95) |
| Comorbidity |  |
| Hypertension | 71,469 (50.06) |
| Diabetes mellitus | 25,583 (17.92) |
| Atrial fibrillation | 2,165 (1.52) |
| Renal disease | 9,044 (6.33) |
| Malignancy | 7,514 (5.26) |
| Premorbid use of medication |  |
| Statin | 20,330 (14.24) |
| Antiplatelet | 14,481 (10.14) |
| Year of RVO index date |  |
| 2008-2011 | 48,348 (33.87) |
| 2012-2015 | 44,339 (31.06) |
| 2016-2020 | 50,072 (35.07) |

The data are represented as numbers (%) or means ± standard deviation.

RVO, retinal vein occlusion.
