# Supplementary material for: Impact of statin treatment on cardiovascular events in patients with retinal vein occlusion: a nested case-control study in Korea
Source: Epidemiol Health. 2023 Mar 15;45:e2023035. doi: 10.4178/epih.e2023035 (PMC10396806; doi:10.4178/epih.e2023035)
Supplement: Supplementary Material 3. — Proportion of patients who had received statins after a diagnosis of retinal vein occlusion [file epih-45-e2023035-Supplementary-3.docx]

**Supplementary Material 3.** Proportion of patients who had received statins after a diagnosis of retinal vein occlusion


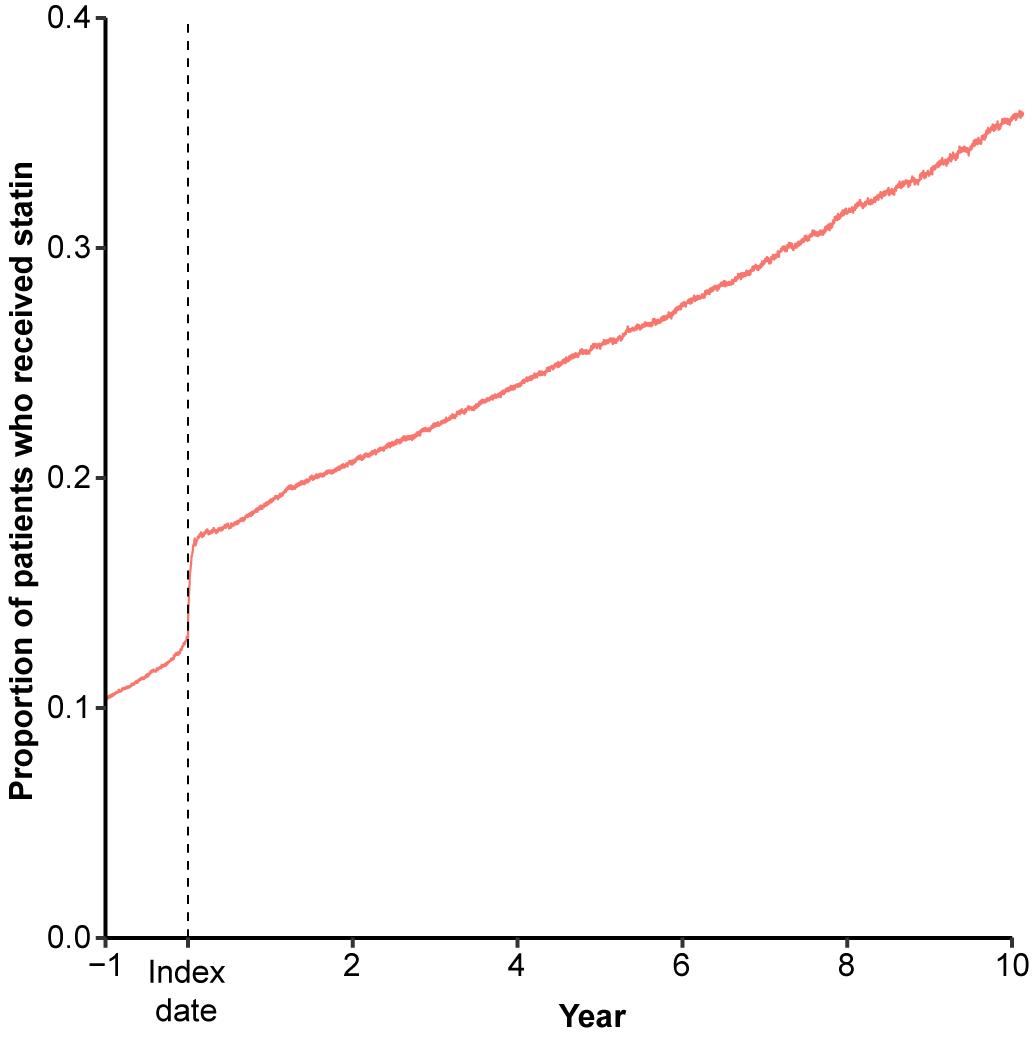


The X-axis indicates the time (year) from the onset of retinal vein occlusion (index date). The Y-axis indicates the proportion of patients who had received the medication among the patients at risk (who were still followed up, excluding the number who died, were included in the outcome, and were censored) at the time point. The range of the X-axis is from 1 year before the diagnosis of retinal vein occlusion to the study end date (the development of stroke, loss of participant eligibility, death, or June 30, 2021, whichever occurred the earliest).
