# Supplementary material for: Impact of statin treatment on cardiovascular events in patients with retinal vein occlusion: a nested case-control study in Korea
Source: Epidemiol Health. 2023 Mar 15;45:e2023035. doi: 10.4178/epih.e2023035 (PMC10396806; doi:10.4178/epih.e2023035)
Supplement: Supplementary Material 4. — Estimates of the cumulative incidence for primary outcome according to the use of statins after retinal vein occlusion [file epih-45-e2023035-Supplementary-4.docx]

**Supplemental Material 4.** Estimates of the cumulative incidence for primary outcome according to the use of statins after retinal vein occlusion


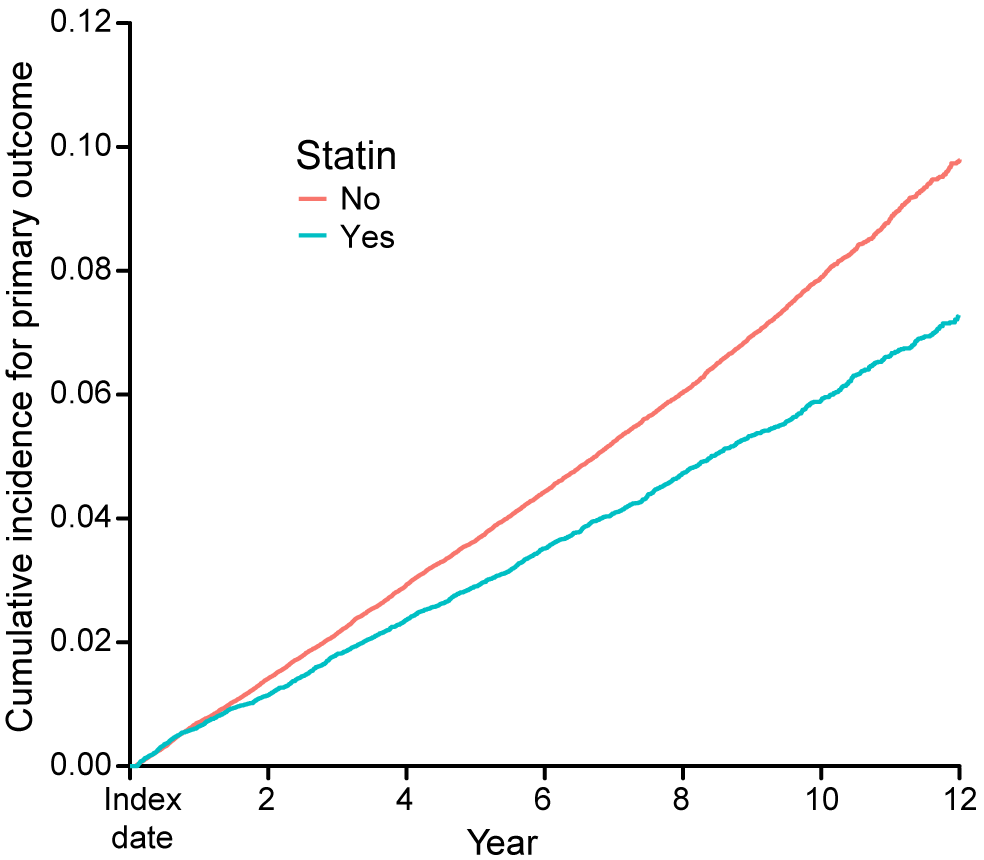


Simon-Makuch plot illustrates the cumulative incidence for primary outcome (stroke and myocardial infarction) according to the use of statins after retinal vein occlusion.
