# Supplementary material for: Impact of statin treatment on cardiovascular events in patients with retinal vein occlusion: a nested case-control study in Korea
Source: Epidemiol Health. 2023 Mar 15;45:e2023035. doi: 10.4178/epih.e2023035 (PMC10396806; doi:10.4178/epih.e2023035)
Supplement: Supplementary Material 5. — Association between statin treatment and cardiovascular risk in subpopulations by year of retinal vein occlusion diagnosis [file epih-45-e2023035-Supplementary-5.docx]

**Supplementary Material 5.** Association between statin treatment and cardiovascular risk in subpopulations by year of retinal vein occlusion diagnosis


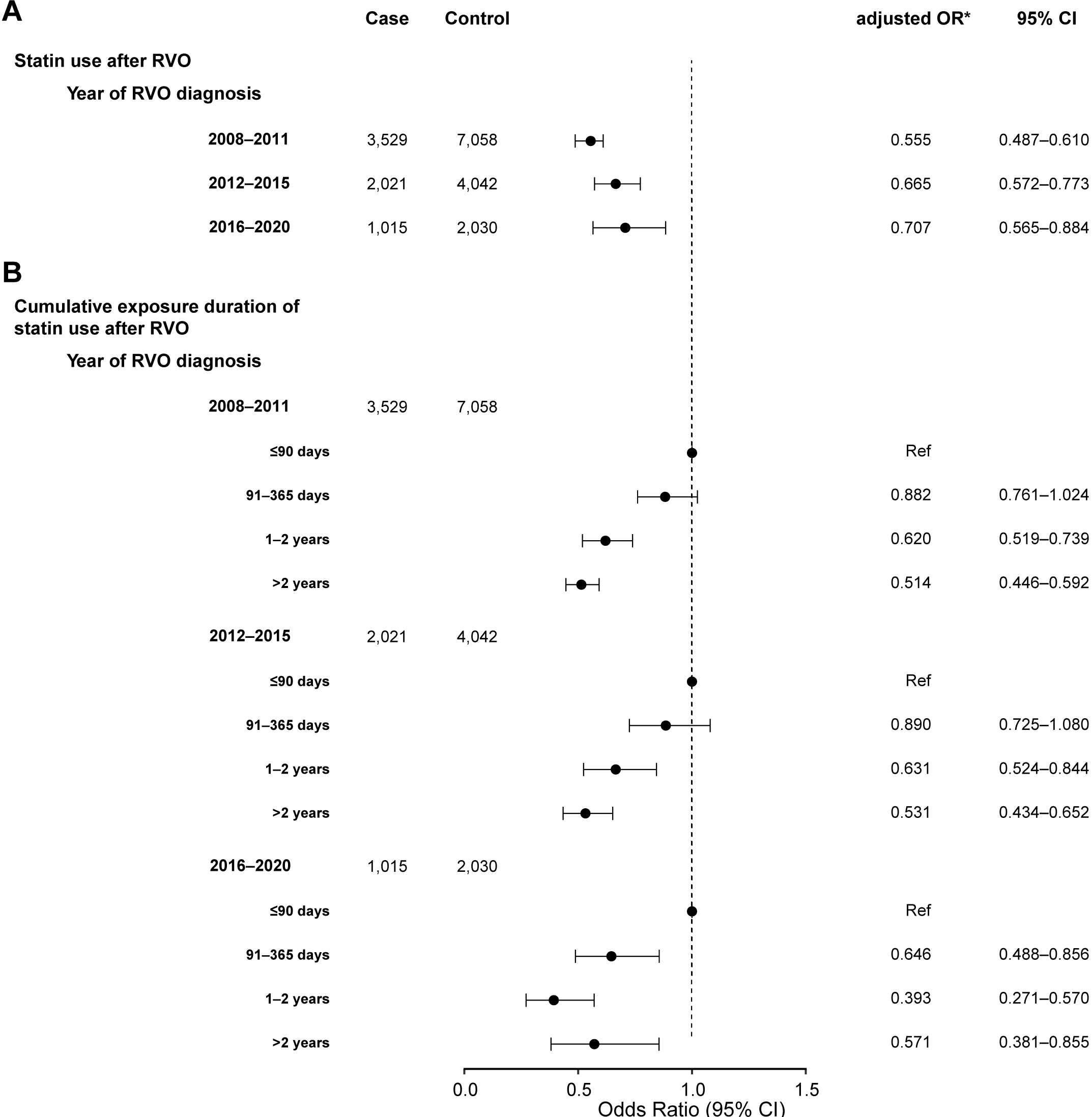


Controls are matched with each case for sex, age, insurance type, hypertension, diabetes mellitus, atrial fibrillation, renal disease, malignancy, premorbid use of antiplatelets before RVO, and treatment with antiplatelets.

^*^Derived from the multivariable conditional logistic regression adjusted for premorbid use of statins. (A) Statin treatment after RVO. (B) Cumulative exposure duration of statin after RVO.

CI, confidence interval; OR, odds ratio; RVO, retinal vein occlusion.
